# Ferroptosis and hepatocellular carcinoma: the emerging role of lncRNAs

**DOI:** 10.3389/fimmu.2024.1424954

**Published:** 2024-05-23

**Authors:** Haoran Chen, Zhongyu Han, Junyan Su, Xuanliang Song, Qingquan Ma, Yumeng Lin, Zijin Ran, Xueping Li, Rongkun Mou, Yi Wang, Dongxuan Li

**Affiliations:** ^1^ Department of General Surgery, Chengdu Xinhua Hospital Affiliated to North Sichuan Medical College, Chengdu, China; ^2^ The First People’s Hospital of Longquanyi District, Chengdu, China; ^3^ School of Basic Medical Sciences, Chengdu University of Traditional Chinese Medicine, Chengdu, China; ^4^ Department of General Surgery, The Third Hospital of Mianyang, Mianyang, China

**Keywords:** hepatocellular carcinoma, cell death, ferroptosis, lipid peroxidation, lncRNA

## Abstract

Hepatocellular carcinoma is the most common form of primary liver cancer and poses a significant challenge to the medical community because of its high mortality rate. In recent years, ferroptosis, a unique form of cell death, has garnered widespread attention. Ferroptosis, which is characterized by iron-dependent lipid peroxidation and mitochondrial alterations, is closely associated with the pathological processes of various diseases, including hepatocellular carcinoma. Long non-coding RNAs (lncRNAs), are a type of functional RNA, and play crucial regulatory roles in a variety of biological processes. In this manuscript, we review the regulatory roles of lncRNAs in the key aspects of ferroptosis, and summarize the research progress on ferroptosis-related lncRNAs in hepatocellular carcinoma.

## 1 Introduction

Cancer has always been a worldwide health problem that perplexes physicians and patients. The American Cancer Society estimates that there will be approximately 2 million new cancer cases and 0.6 million cancer-related deaths in the United States in 2024 (1). With advances in medical treatment, the overall 5-year survival rate of cancer patients has increased from 49% in the 1970s to 69% in the 2010s. However, the survival rate of patients with liver cancer remains low at 22% (1). Hepatocellular carcinoma (HCC) is the main form of primary liver cancer, accounting for 90% of liver cancers (2, 3). The molecular mechanism of HCC is complex and is related to susceptibility genes and viral and nonviral risk factors. Common risk factors for HCC include hepatitis B virus (HBV), hepatitis C virus, and fatty liver disease, which can cause chronic hepatitis and lead to cirrhosis and eventually to HCC (4). Moreover, the persistence of cirrhosis leads to a high recurrence rate of HCC (2). Despite significant advances in the treatment and management of HCC, the overall prognosis of patients remains unsatisfactory. Precisely targeted therapies are required to eliminate tumor cells and minimize side effects on healthy cells.

Killing tumor cells is an important part of tumor treatment. In multicellular organisms, cell death is an important mechanism that helps maintain the cell balance while renewing cells. Cell death can be divided into programmed cell death (PCD) and accidental cell death, and PCD can be classified as apoptosis, necroptosis, autophagy, pyroptosis, ferroptosis, or entosis (5). Ferroptosis is a type of PCD that is characterized by iron-dependent lipid peroxidation. Morphologically, when cells undergo ferroptosis, mitochondria exhibit shrinkage, reduced numbers of cristae, and an increased membrane density (6). Mitochondria are important organelles involved in energy metabolism and biosynthesis. Reactive oxygen species (ROS) are intermediate products of mitochondrial respiration and are highly oxidative. Mitochondria of cancer cells characteristically produce more ROS, which facilitate the progression of cancer by promoting the expression of oncogenes and related signaling pathways (7–9). Compared with that in healthy cells, iron metabolism in cancer cells is often altered towards iron accumulation, and iron overload promotes cancer development (10). Therefore, targeting cancer cell ferroptosis may be a potential cancer therapy with few side effects.

Non-coding RNA (ncRNA) refers to RNA that is transcribed but is not translated, and ncRNAs account for 98–99% of the eukaryotic genome (11). Although they do not encode proteins, ncRNAs affect the expression of genes and proteins in different ways and participate in various physiological and pathological processes. Long non-coding RNAs (lncRNAs) are the largest subgroup of ncRNAs with lengths >200 nt. At present, tens of thousands of lncRNAs have been identified and divided into the following five categories: sense, antisense, bidirectional, intergenic, and intronic lncRNAs (12, 13). Recent studies have shown that lncRNAs are involved in the regulation of iron overload, ROS production, and other important ferroptosis-related processes (14–16). Therefore, targeting ferroptosis-related lncRNAs may be a promising therapeutic approach for treating cancers such as HCC.

In this manuscript, we review the research progress on the key links and mechanisms of ferroptosis, summarize the regulatory effects of lncRNAs on ferroptosis, and explore the potential mechanisms of lncRNA-mediated ferroptosis and their significance in HCC. We believe that this review will help further understand the role of ferroptosis-related lncRNAs in HCC and provide novel strategies for the treatment of this malignancy.

## 2 Ferroptosis and HCC

Ferroptosis was initially discovered as a unique form of cell death that was induced by erastin and RSL3 in RAS-expressing cancer cells; this type of cell death did not have the characteristics of apoptosis, nor could it be reversed by caspase inhibitors (17). The term ‘ferroptosis’ was first used in 2012 by Dixon et al. to describe a form of cell death that was characterized by intracellular iron dependency and was distinct from other types of PCD; the term was finally recommended by the Nomenclature Committee on Cell Death (NCCD) in 2018 (18, 19). Mechanistically, ferroptosis is triggered by iron-dependent lethal levels of lipid peroxidation, resulting in characteristic mitochondrial changes and necrosis-like changes in cells (17). The solute carrier family 7 member 11 (SLC7A11)/glutathione (GSH)/glutathione peroxidase 4 (GPX4), ferroptosis suppressor protein 1 (FSP1)/CoQ_10_H_2_ and GTP cyclohydrolase-1 (GCH1)/tetrahydrobiopterin (BH_4_) axes constitute the main intracellular ferroptosis defense system and inhibit lipid peroxidation through strong reducing products (20). When these ferroptosis defense axes are impaired, intracellular iron overload leads to the generation of large amounts of ROS via the Fenton reaction, which promotes lipid peroxidation and ferroptosis (21). In addition, the phosphatidylinositol 3-kinase (PI3K)/protein kinase B (Akt)/mammalian target of rapamycin (mTOR), p53, AMPK, nuclear factor E2-related factor 2 (Nrf2)/HO-1, hypoxia, and other signaling pathways are involved in the regulation of ferroptosis (6).

**Figure 1:**
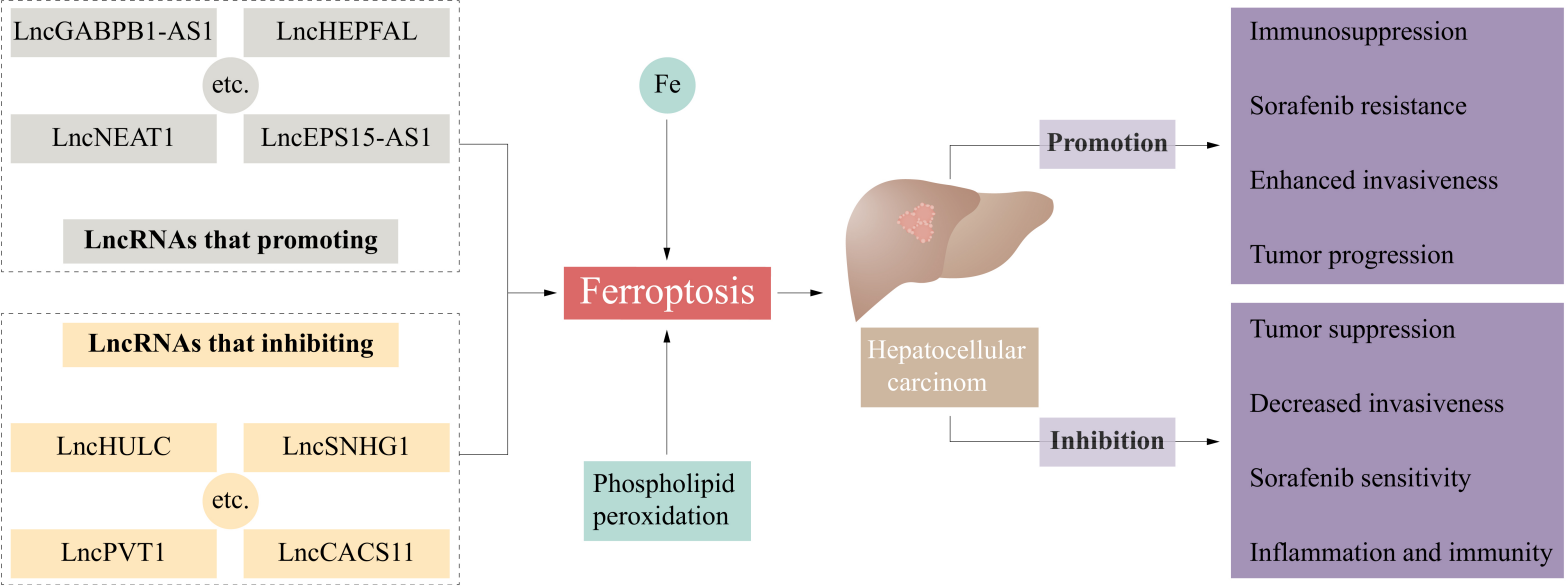
Ferroptosis-related lncRNAs influence the occurrence and development of hepatocellular carcinoma. CASC11, cancer susceptibility candidate 11; EPS15, epidermal growth factor receptor pathway substrate 15; GABPB1, GA-binding protein B1; HULC, highly upregulated in liver cancer; LncRNA, long non-coding RNA; PVT1, plasmacytoma variant 1; SNHG1, small nucleolar RNA host gene 1.

The liver is the primary site for iron storage and metabolism, as well as an essential organ for lipid and amino acid metabolism (22). In the liver, excessive iron storage or metabolic disorders can lead to abnormally high iron levels within cells and an increased production of ROS. Abnormal lipid metabolism may cause excessive accumulation of lipids within cells, creating a lipotoxic environment (23). Meanwhile, abnormal amino acid metabolism can affect the synthesis of antioxidants such as glutathione, reducing the cell’s resistance to oxidative stress. Consequently, this high metabolic state makes hepatocytes prone to ferroptosis under specific conditions, contributing to the development of various liver diseases. In recent years, increasing number of studies have shown that ferroptosis plays a role in the occurrence, progression, and treatment of HCC. Iron overload is a risk factor for HCC (24). Liang et al. analyzed TCGA cohort data of HCC patients and showed that more than 80% of ferroptosis-related genes were differentially expressed between HCC tissues and peritumoral tissues, and a prediction model including 10 ferroptosis-related genes such as *GPX4* and *SLC7A11* was established to predict the prognosis of HCC patients (25). Ferroptosis is an important mechanism of radiotherapy-induced tumor cell death (5). The regulation of important factors involved in ferroptosis (such as SLC7A11) can promote ferroptosis and ionizing radiation sensitization in HCC cells (26, 27). Currently, the first-line chemotherapy agents for HCC are sorafenib and lenvatinib (4). Sorafenib, a multiple-target tyrosine kinase inhibitor, has been shown to induce ferroptosis in various solid tumors, such as HCC, melanoma, and colon carcinoma (28–30). The tyrosine kinase inhibitor lenvatinib is noninferior to sorafenib in treating HCC (4, 31). Lenvatinib can inhibit SLC7A11 and GPX4 by inhibiting fibroblast growth factor receptor-4 (FGFR4), leading to ferroptosis in HCC cells (32). Ferroptosis is also closely related to immunotherapy for HCC. Immunotherapy aims to change the tumor microenvironment (TME) and inhibit tumor progression. Liu et al. established a ferroptosis and epithelial-mesenchymal transition (EMT)-related prognostic model (FEPM) to predict the prognosis of HCC patients. According to this model, the expression of ferroptosis suppressor genes such as *GPX4*, *SLC7A11*, *FTH1*, and *SCD*, was increased in the high-FEPM group, and immunosuppressive cells such as Tregs were highly enriched, resulting in a worse prognosis of patients (33). In another study, KEGG pathway analysis revealed that erastin-induced signaling cascade could affect Th17 cell differentiation and IL-17 signaling pathway in HCC (34). Single-cell RNA sequencing revealed that APOC1^+^ macrophages were abundant in HCC tissues, and expressed the M2 macrophage marker CD163, which plays a tumor-promoting and immunosuppressive role (35). APOC1 inhibition can promote the transformation of tumor-associated macrophages (TAMs) from the M2 to the M1 phenotype through the ferroptosis pathway. APOC1 inhibition was shown to reduce the expression of GPX4, SLC7A11, and Nrf2, and iron overload-induced ROS could also promote M1 polarization and inhibit HCC (36). Based on the important role of ferroptosis in the occurrence, progression, and treatment of HCC, targeting ferroptosis could be a potential direction for HCC treatment. In the following subsections, we discuss the effects of lncRNAs on ferroptosis at different stages and the function of ferroptosis-associated lncRNAs in HCC (**Figure 1**).

## 3 LncRNAs and ferroptosis

### 3.1 LncRNAs and iron metabolism

**Figure 2:**
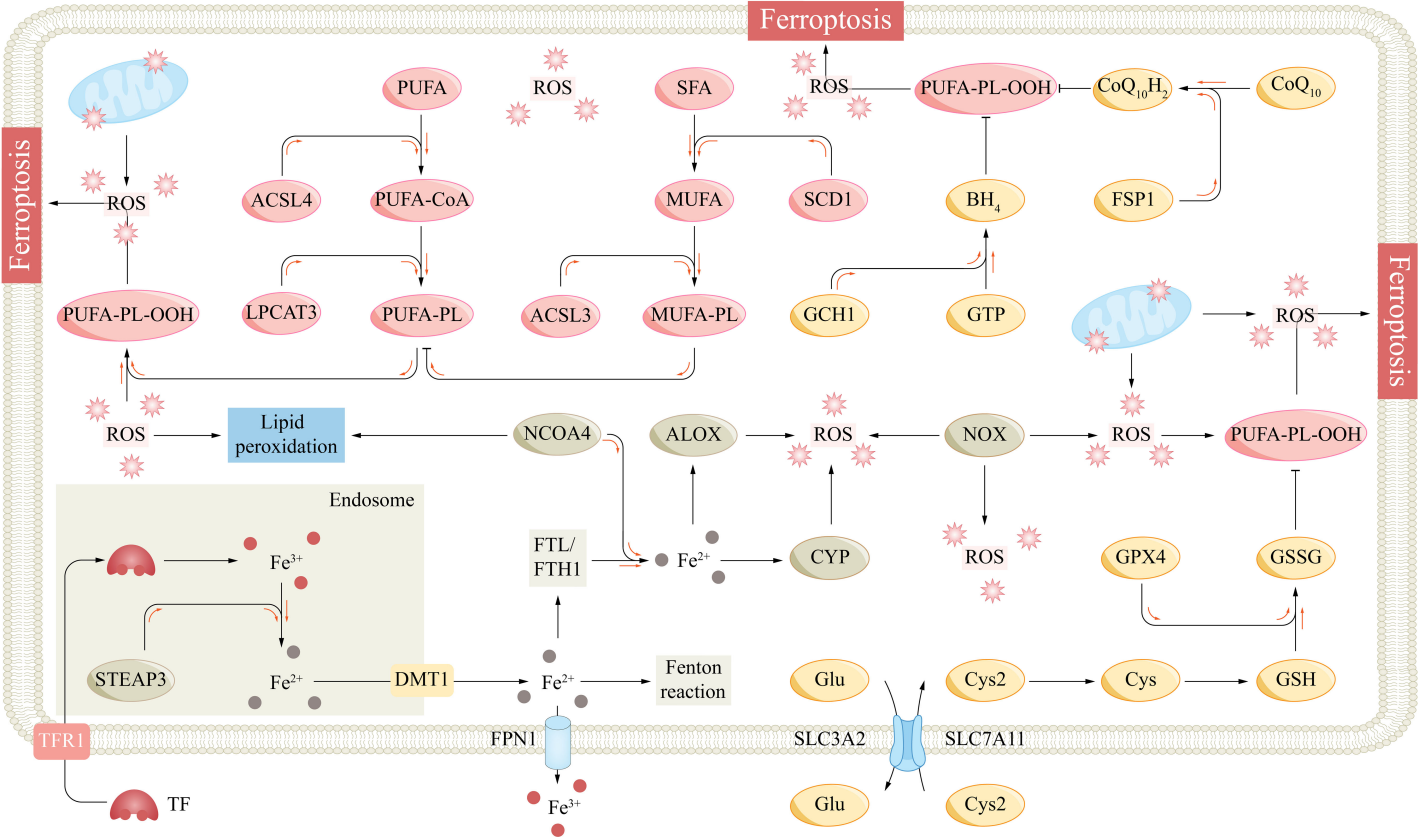
The fundamental mechanism underlying ferroptosis. ACSL3/4, acyl-CoA synthetase long-chain family member 3/4; ALOX, arachidonate lipoxygenase; BH4, tetrahydrobiopterin; CoQ10, coenzyme Q10; CYP, cytochrome p450 family; Cys, cysteine; Cys2, cysteine; DMT1, divalent metal transporter 1; Fe^2+^, ferrous; Fe^3+^, ferric; FPN1, ferroportin 1; FSP1, ferroptosis suppressor protein 1; FTH1, ferritin heavy chain 1; FTL, ferritin light chain; GCH1, GTP cyclohydrolase-1; Glu, glutamate; GPX4, glutathione peroxidase 4; GSH, glutathione; GSSG, glutathione disulfide; GTP, guanosine triphosphate; LPCAT3, lysophosphatidylcholine acyltransferase 3; MUFA, monounsaturated fatty acid; NCOA4, nuclear receptor coactivator 4; NOX, NADPH oxidase; PL, phospholipid; PLOO, phospholipid peroxyl radical; PUFA, polyunsaturated fatty acid; ROS, reactive oxygen species; SCD1, stearoylCoA desaturase-1; SFA, saturated fatty acid; SLC7A11, subunit solute carrier family 7 member 11; STEAP3, six transmembrane epithelial antigen of the prostate 3; TF, transferrin; TFR1, transferrin receptor 1.

Iron is an essential trace element for human body and participates in a wide range of biological processes, such as cellular respiration, energy metabolism, oxygen transport, and DNA synthesis (10). Normal intracellular iron metabolism maintains intracellular iron homeostasis and cell function. Cells acquire transferrin (TF)-bound ferric (Fe^3+^) iron from the circulation through a transferrin receptor (TFR1). Ferric iron is subsequently reduced to ferrous (Fe^2+^) iron in endosomes by metalloreductases, such as six-transmembrane epithelial antigen of prostate 3 (STEAP3), and is transported to the cytoplasm via divalent metal transporter 1 (DMT1) to participate in intracellular biological processes (37). Fe^2+^ that is not involved in biological processes is excreted from the cell by ferroportin 1 (FPN1) or binds to ferritin for storage. FPN1 is the only known intracellular iron exporter, and hepcidin is a major regulator of FPN1 (38). Excess intracellular Fe^2+^ can be oxidized by ferritin to Fe^3+^, and Fe^3+^ is stored in a complex with ferritin light chain (FTL) or ferritin heavy chain 1 (FTH1) (30). Nuclear receptor coactivator 4 (NCOA4) can bind to ferritin, promote ferritinophagy, and release iron stored in ferritin (39). Abnormal iron metabolism can lead to increased intracellular free iron levels and iron overload (**Figure 2**).

Lu et al. reported that the lncRNA plasmacytoma variant 1 (PVT1) could inhibit miR-214, and in acute ischemic stroke (AIS) with cerebral ischemia/reperfusion (I/R) injury, PVT1 silencing could promote miR-214 expression, suppress TFR1 and ferritin levels, and reduce ferroptosis (40). Mechanistically, miR-214 could bind to PVT1 and TFR1, and PVT1 silencing promoted the binding of miR-214 to the 3’ untranslated region (3’UTR) of TFR1 and reduced its expression. He et al. reported that the lncRNA related to iron metabolism (LncRIM) could directly bind to neurofibromatosis 2 (NF2), activate the Hippo pathway and activate Yes-associated protein 1 (YAP1) to promote the expression of TFR1 and DMT1 and to promote breast cancer cell ferroptosis (41). Studies have shown that FPN1 has an iron chaperone protein poly(rC)-binding protein 2 (PCBP2)-binding domain and that iron-loaded PCBP2 binds to FPN1 to promote intracellular iron export (42). Xiang et al. reported that the lncRNA MAF transcription factor G antisense RNA 1 (MAFG-AS1) could promote PCBP2 stabilization and iron export in bladder urothelial carcinoma (BUC) cells (43). MAFG-AS1 expression is regulated by the transcription factor MAFG and can also promote MAFG transcription via positive feedback. MAFG-AS1-MAFG inhibition promotes ferroptosis and sensitivity to cisplatin in BUC cells.

### 3.2 LncRNAs and lipid peroxidation

Phospholipids (PLs) are one of the important components of the cell membrane and are composed of a head group and two fatty acyl chains. Different fatty acyl chains, such as saturated fatty acids (SFAs), monounsaturated fatty acids (MUFAs), and polyunsaturated fatty acids (PUFAs), link the head group at the *sn1* and *sn2* sites, enriching phospholipid diversity (44). Free PUFAs, such as epinephrine and arachidonic acid, are processed by acyl-coenzyme A synthetase long-chain family member 4 (ACSL4), lysophosphatidylcholine acyltransferase 3 (LPCAT3), and other enzymes to bind to the *sn2* site to form PUFA-PLs and are closely associated with the fluidity of cell membranes (45). However, PUFAs are lipids that are most susceptible to peroxidation, which leads to ferroptosis. Lipid peroxidation is caused by strong oxidants that attack the carbon–carbon double bonds (C=C) of lipids (46). PUFA-PLs contain bis-allylic groups and are easily oxidized to form phospholipid radicals (PL•), which subsequently form phospholipid peroxyl radicals (PLOO•) with oxygen molecules (20). Importantly, PLOO• can seize a hydrogen atom from the bis-allylic group of another PUFA-PL, leading to the formation of another PL• and the transmission of lipid peroxidation (47). Under iron overload conditions, a large amount of ROS produced by the Fenton reaction induce nonenzymatic peroxidation of PUFAs (48). Some iron-dependent oxygenases, such as arachidonate lipoxygenase (ALOX) and cytochrome P450 (CYP) family proteins, can cause lipid peroxidation (**Figure 2**) (49, 50).

Several lncRNAs have been shown to regulate ACSL4. In high-glucose-induced diabetic retinopathy, the highly expressed lncRNA zinc finger antisense RNA 1 (ZFAS1) promoted ferroptosis in human retinal endothelial cells (hRECs). ZFAS1 acted as a sponge for miR-7–5p to competitively regulate the expression of ACSL4 and drive lipid peroxidation in hRECs (51). Sun et al. reported that the lncRNA taurine-upregulated gene 1 (TUG1) interacted with serine/arginine splicing factor 1 (SRSF1) to regulate ACSL4 mRNA stability and inhibit ACSL4-mediated ferroptosis and acute kidney injury (52). Jin et al. reported that the lncRNA HOX transcript antisense RNA (HOTAIR) could directly bind to up-frameshift 1 (UPF1), competitively inhibit UPF1-ACSL4 binding and ACSL4 degradation, and positively regulate ACSL4-dependent neuronal ferroptosis (53). LncRNA small nucleolar RNA host gene 1 (SNHG1) knockdown was shown to regulate the miR-16–5p/ACSL4 axis to suppress hyperglycemia-induced ferroptosis in diabetic nephropathy (54).

Stearoyl-CoA desaturase 1 (SCD1) is a key enzyme of lipid metabolism that is capable of catalyzing the conversion of SFAs into MUFAs. SCD1-catalyzed MUFAs can replace PUFAs and inhibit ferroptosis in an ACSL3-dependent manner (55). Luo et al. reported that the lncRNA LINC01606 was highly expressed in colon cancer and regulated SCD1 expression through competitive inhibition of miR-423–5p, which promoted MUFA production and ferroptosis inhibition (56). In addition, the positive feedback loop of SCD1–Wnt/β-catenin–IGHM enhancer 3 (TFE3) signaling promoted LINC01606 expression, which continuously increased the MUFA levels and protected colon cells against ferroptosis. Lai et al. reported that the exosomal lncRNA SRY-box transcription factor 2 overlapping transcript (SOX2-OT) could promote the progression of ovarian cancer cells through the miR-181b-5p/SCD1 axis (57).

### 3.3 LncRNAs and the ferroptosis defense system

Under physiological conditions, cells inhibit lipid peroxidation through several antioxidant systems. The subunit solute carrier family 7 member 11 (SLC7A11)/glutathione (GSH)/glutathione peroxidase 4 (GPX4) axis is a major ferroptosis defense system, and its inactivation can lead to severe ferroptosis (58). GPX4 is the major lipid peroxide reductase that reduces PL-OOH to nontoxic PL-OH, and its activity is dependent on GSH and selenium (59). GSH is a tripeptide composed of glutamate, glycine, and cysteine and is the main endogenous nonprotein antioxidant. GSH is reduced to oxidized glutathione (GSSG) by GPX4 to drive the reduction of PL-OOH, and this process is inhibited when the GSH concentration is insufficient. Cysteine is the major rate-limiting precursor for GSH synthesis, and most cells need to obtain cysteine by extracellular transport, except for tissues (such as the liver) that can synthesize cysteine through the transsulfuration pathway (60). Extracellular cysteine is transported by the cystine/glutamate reverse transporter (X_C_-). Its heavy chain SLC7A11 transports extracellular cystine and excretes intracellular glutamate at a 1:1 ratio; subsequently, cystine is reduced to cysteine by NADPH and participates in GSH synthesis (58, 61). The SLC7A11-GSH-GPX4 axis constitutes a major PL-OOH detoxification system (**Figure 2**).

Various lncRNAs have been demonstrated to regulate the SLC7A11/GSH/GPX4 axis. The lncRNA T-UCR Uc.339 was shown to inhibit miR-339 and promote SLC7A11 expression, also promoting lung adenocarcinoma growth and metastasis (62). The lncRNA HEPFAL can regulate the ubiquitination of SLC7A11 and affect its function (63). The lncRNA double homeobox A pseudogene 8 (DUXAP8) can regulate SLC7A11 palmitoylation and protect HCC cells (64). The lncRNA ovarian tumor domain containing 6B antisense RNA1 (OTUD6B-AS1) can promote GPX4-mediated ferroptosis and increase the radiosensitivity of colorectal cancer cells (65). The lncRNA LINC01134 inhibits ferroptosis and promotes oxaliplatin resistance of HCC by promoting Nrf2-GPX4 binding (66). The lncPVT1/miR-214 axis can also promote GPX4 expression and the proliferation of liver cancer cells (67).

The ferroptosis suppressor protein 1 (FSP1)/ubiquitin (CoQ_10_) and GTP cyclohydrolase-1 (GCH1)/tetrahydrobiopterin (BH_4_) axes defend cells against ferroptosis in parallel with the SLC7A11/GSH/GPX4 axis. CoQ_10_ is synthesized in mitochondria and is involved in electron transport in the mitochondrial respiratory chain (68). CoQ_10_ can be transformed into oxidized and reduced states, and its fully reduced form, CoQ_10_H_2_, has strong antioxidant activity. FSP1 is a NAD(P)H-dependent CoQ_10_ reductase, and the FSP1/CoQ_10_ axis consumes NAD(P)H and promotes the reduction of CoQ_10_ to CoQ_10_H_2_, which directly consumes lipid free radicals (69). BH_4_ also has strong antioxidant properties and can directly reduce lipid free radicals. GCH1 can catalyze the conversion of guanosine triphosphate (GTP) to BH_4_ (70). Dihydroorotate dehydrogenase (DHODH) can also couple the oxidation of dihydroorotate (DHO) to orotate (OA) to reduce CoQ_10_ to CoQ_10_H_2_ in the mitochondrial inner membrane (71). Mao et al. reported that DHODH could inhibit ferroptosis in parallel with GPX4 and FSP1 (71). However, Mishima et al. reported that the ferroptosis effect of DHODH appeared to be small and environment dependent, with high concentrations of DHODH inhibitors also inhibiting FSP1 and inducing ferroptosis mainly through FSP1 inhibition (**Figure 2**) (72).

LINC02587 expression is elevated in various cancers and is linked to a poor prognosis (73–75). Wang et al. reported that LINC02587 silencing led to the inhibition of the CoQ10/FSP1 axis and ferroptosis promotion in glioma cells (75). Similarly, high expression of LINC01133 is associated with a poor prognosis in cancers (76). Wang et al. reported that overexpression/knockdown of LINC01133 resulted in increased/decreased expression of FSP1, respectively, but did not affect that of GPX4. An RNA pulldown assay revealed that LINC01133 and FSP1 could bind to FUS to form the LINC01133-FUS-FSP1 complex, which regulated the expression of FSP1 mRNA (77). There are no reports on lncRNA regulation of the GCH1/BH_4_ axis, and this is a potential research direction. The circRNA circLRFN5 was shown to suppress paired related homeobox 2 (PRRX2)-mediated GCH1 expression in glioblastoma (78). A study also reported that the lncRNA USP30−AS1 was coexpressed with GCH1 in breast cancer (79).

### 3.4 LncRNAs and mitochondria

Mitochondrion is an important organelle of eukaryote, which is involved in biological processes such as energy production, material metabolism, signal transduction, etc (80). Mitochondria play important roles in ferroptosis. Mitochondria are the center of iron metabolism and iron balance, participating in the storage, transportation, and excretion of iron within cells. Iron within mitochondria is involved in the formation of iron-sulfur clusters, heme, and mitochondria ROS (mitoROS) (81). As the core of the iron-porphyrin structure, iron is a crucial component of enzymes such as catalase and peroxidase. Iron ions participate in the electron transport of the mitochondrial respiratory chain, promoting the progress of redox reactions (82). Intracellular ROS are mainly produced by mitochondria. The tricarboxylic acid (TCA) cycle is a key metabolic pathway in mitochondria, connecting the metabolism of glucose, lipid, and amino acids, and providing energy for cells (83). Changes in the levels of certain intermediates in the TCA cycle, such as isocitrate and 2-oxoglutarate, can affect the accumulation of lipid ROS and the progress of ferroptosis (84). The electron transport chain (ETC) is a series of complexes located on the inner membrane of mitochondria, which generate ATP by transferring electrons, providing energy for cells, and this process produces ROS (84). Additionally, there is a free iron pool within mitochondria that participates in the accumulation of mitoROS (85). Functionally normal mitochondria can also resist oxidative stress and lipid peroxidation through antioxidant substances. The GSH-GPX4 axis within mitochondria plays an important antioxidant role. In addition, dihydroorotate dehydrogenase (DHODH) is an antioxidant parallel to GPX4, which reduces CoQ_10_ to CoQ_10_H_2_ and can inhibit ferroptosis when GPX4 is inactivated (71). However, another study showed that the inhibitory effect of DHODH on ferroptosis is minimal and is related to FSP1 (72). Finally, mitophagy can alleviate intracellular oxidative stress and inhibit ferroptosis by removing aged or damaged mitochondria (86).

Various lncRNAs play important roles in the regulation of mitochondrial function. A study based on public databases has identified eight lncRNAs (AC023090.1, AC099850.4, AL365361.1, CYTOR, ACSL6-AS1, LHFPL3-AS2, LINC02362, and MSC-AS1) that potentially affect mitochondrial function and ferroptosis in HCC through cell cycle pathways (87). Zhao et al. found that the lncRNA metastasis-associated lung adenocarcinoma transcript 1 (MALAT1) plays a role in mitochondrial gene reprogramming in HCC. RNA reverse transcription-associated trap sequencing (RAT-seq) revealed that MALAT1 interacts with multiple sites on mitochondrial DNA. MALAT1 function as an epigenetic messenger to coordinate the functions between the nucleus and mitochondria. Although they did not examine ferroptosis-related indicators, knocking down MALAT1 can inhibit mitochondrial autophagy and induce apoptosis (88).

## 4 Ferroptosis-related lncRNAs and HCC

As potential biomarkers, lncRNAs play crucial roles in the genesis, progression, and metastasis of HCC, which renders them viable options for assessing tumor prognosis based on their expression. In recent years, several ferroptosis-related lncRNA models have been constructed to predict the prognosis of patients with HCC (87, 89–97). Nevertheless, predictive models, particularly those based on RNA-sequencing and retrospective bioinformatics data analysis are based on retrospective bioinformatics data analyses and necessitate additional experimental validation to ensure their reliability. Xu et al. established a ferroptosis-related 9-lncRNA model (LINC00942, LINC01224, LINC01231, LINC01508, CTD-2033A16.3, CTD-2116N20.1, CTD-2510F5.4, DDX11-AS1, and ZFPM2-AS1) to predict HCC prognosis and the immune response, and the expression of these lncRNAs was increased in the high-risk group, which was correlated with the suppression of HCC cell ferroptosis (98). CTD-2033A16.3, LINC01231, and LINC01508 knockdown led to increased intracellular Fe^2+^ and malondialdehyde (MDA) levels in the HCC cell line HUH-7. In the high-risk group, the numbers of Tregs, follicular helper T cells, and M0 macrophages were increased, and the immune tolerance of HCC patients was enhanced, which provided potential targets for immunotherapy in HCC patients. Xie et al. established a model that comprised 16 m6A-methylated and ferroptosis-associated lncRNAs (99). RNA N6-methyladenosine (m6A) methylation modification specifically targets the sixth nitrogen of adenine (100). m6A plays a crucial role in the splicing, transport, stability, and degradation of ncRNAs and is strongly associated with tumorigenesis and ferroptosis (101, 102). These lncRNAs are associated with ferroptosis and are methylated at m6A, which significantly increases the utility of the model in accurately predicting the clinical stage, prognosis, immune infiltration, and hot and cold tumor subtypes in HCC. A study based on multiomics data revealed that the lncRNA MALAT1 was highly expressed in HCC and was associated with various tumor signatures, including high cell cycle, DNA damage repair, mismatch repair, and homologous recombination signature feature scores and a low ferroptosis feature score (103). Although knocking down MALAT1 has been shown to induce ferroptosis in endometriosis, there is currently no research exploring the mechanism by which MALAT1 regulates ferroptosis in HCC (104).

PVT1 is upregulated in various cancers and promotes cancer progression (105). He et al. reported that PVT1 acted as a competing endogenous RNA (ceRNA), competitively binding to miR-214–3p, inhibiting its binding to GXP4, and promoting GPX4 expression. Ketamine treatment inhibited the expression of PVT1 and GPX4 and induced ferroptosis in HCC cells (67). Similarly, the lncRNA HLA complex group 18 (HCG18) is overexpressed in HCC, particularly in sorafenib-resistant HCC cells (106, 107). As a ceRNA, HCG18 suppressed miR-450b-5p and concomitantly increased the expression of GPX4. Inhibition of HCG18 enhanced the accumulation of MDA and ROS in HCC cells, thereby increasing their sensitivity to sorafenib (106). Chen et al. discovered that high expression of the lncRNA cancer susceptibility candidate 11 (CASC11) in HCC contributed to its resistance to sorafenib. CASC11 directly interacted with SLC7A11 mRNA, increasing its stability and half-life. CASC11 silencing resulted in the elevation of Fe^2+^, ROS, and MDA levels in HCC cells, which potentiated the therapeutic impact of sorafenib (108). LncRNA NRAV is highly expressed in different HCC cell lines, affecting the expression of GPX4, SLC7A11, and ACSL4 and inhibiting ferroptosis. Functional experiments revealed that NRAV can bind to miR-375–3p, promoting the expression of SLC7A11. However, the specific mechanism remains unclear (109). Zhang et al. reported that the expression of HEPFAL in HCC was significantly reduced, and HCC cells with lower HEPFAL expression had greater invasion and migration abilities. Overexpression of HEPFAL in HCC cells increased the intracellular Fe^2+^ and ROS levels and the sensitivity of HCC cells to erastin-induced ferroptosis (63). Mechanistically, HEPFAL overexpression led to the ubiquitination of SLC7A11, reducing its stability and half-life, but had no significant effect on FSP1. In addition, HEPFAL may inhibit the PI3K/Akt/mTOR signaling pathway, thereby inhibiting GPX4 expression. In another study, lncRNA sequencing revealed a significant increase in the expression of URB-AS1 in HCC tissues, which was positively correlated with the tumor size, grade, and resistance to sorafenib (110). Sorafenib-resistant HCC cells overexpressed URB-AS1 and exhibited decreased levels of free iron, ROS, and lipid peroxidation. Under hypoxic conditions, hypoxia-inducible factor-1α (HIF-1α) is activated, and URB-AS1 is upregulated. URB-AS1 colocalized and interacted with ferritin in the cytoplasm, promoting its punctate distribution and liquid−liquid phase separation. This led to the inhibition of NCOA4-mediated ferritinophagy and a decrease in the level of free iron. Long-term sorafenib treatment may induce hypoxia within the HCC tumor microenvironment by decreasing microvascular density, which leads to the activation of HIF-1α (111). This HIF-1α activation may enhance the resistance to ferroptosis and sorafenib by activating the URB-AS1 promoter. Yuan et al. reported the regulatory effect of the lncRNA ferroptosis-associated lncRNA (lncFAL, NONHSAG111059.1) on the FSP1/CoQ_10_ pathway (112). LncFAL originates from the plexin B2 (PLXNB2) gene and is modified by YTH N6-methyladenosine RNA-binding protein 2 (YTHDF2) from pre-lncFAL in an m6A-dependent manner. High-density lipoprotein-binding protein (HDLBP) binds to lncFAL and promotes its stabilization. Stable lncFAL remains inactive in regulating the expression of GPX4 or FSP1 mRNA, yet it effectively competes with Trim69 to hinder its binding to FSP1. This competitive inhibition suppresses the ubiquitination-mediated degradation of FSP1 and ferroptosis in HCC cells.

Nrf2 is a crucial transcription factor in regulating cellular antioxidant responses (113). Under physiological conditions, Nrf2 binds to its negative regulator Kelch-like ECH-associated protein 1 (Keap1), which makes Nrf2 inactive, and intracellular Nrf2 is maintained at low levels. However, during oxidative stress, Nrf2 dissociates from Keap1 and binds to the antioxidant response element (ARE) in the nucleus, thereby activating diverse downstream antioxidant pathways (114). Nrf2 plays a pivotal role in regulating various ferroptosis-related factors, including GPX4, SLC7A11, and intracellular free iron (58). The overexpression of the lncRNA LINC01134 is positively associated with a poor prognosis in HCC patients. Mechanistically, LINC01134 facilitates the recruitment of Nrf2 to the GPX4 promoter, which leads to the upregulation of GPX4 expression and the development of resistance to oxaliplatin (66). Peroxiredoxin 5 (PRDX5) is a nonselenium-dependent peroxidase that has been demonstrated to be a binding partner of Nrf2 (115). GABPB1 serves as an activating subunit of Nrf2, and the promoter region of PRDX5 contains binding sites for GA-binding protein (GABP) (116). Overexpression of GABPB1 in HCC cells attenuates ROS and MDA levels, protecting cells from ferroptosis. GABPB1-AS1 is the antisense lncRNA of GABPB1. Qi et al. discovered that erastin treatment potentiated the expression of GABPB1-AS1 and prolonged its half-life. Consequently, GABPB1-AS1-caused suppression of GABPB1 translation resulted in reduced expression of PRDX5 and triggered ferroptosis in HCC cells (117).

Fanconi anemia complementation group D2 (FANCD2), a pivotal nuclear protein that is integral to DNA damage repair, serves as a critical negative regulator of ferroptosis by modulating iron metabolism and lipid oxidation, thereby protecting cells from ferroptosis-induced death (118, 119). A comprehensive database analysis demonstrated that FANCD2 was overexpressed in various tumors, including HCC, and exhibited a positive correlation with immune cell infiltration (CD4^+^ and CD8^+^ T cells, B cells, macrophages, etc.) and immune checkpoints (PD-L1, CTLA-4, etc.) (120). Upstream lncRNA and miRNA analysis suggested that LINC00511 and particularly DUXAP8 might function as ceRNAs in the miR-29c-3p/FANCD2 axis, promoting FANCD2 expression and contributing to HCC progression. Another study showed that DUXAP8 was significantly elevated in HCC tissues, was positively correlated with SLC7A11 levels, and was associated with a poor prognosis in patients with HCC. DUXAP8 silencing led to the inhibition of HCC progression and an increase in its sensitivity to sorafenib. DUXAP8 can promote the palmitoylation of SLC7A11 and enhance its stability through the lysosomal pathway (64). Glucose-6-phosphate dehydrogenase (G6PD) is a crucial enzyme within the pentose phosphate pathway that plays a pivotal role in the promotion of NADPH production and the preservation of cellular redox homeostasis (121). SNHG1 serves as a ceRNA and is overexpression in HCC cells, effectively sponging miR-199a-5p/3p and subsequently upregulating FANCD2 and G6PD, which ultimately leads to the inhibition of ferroptosis (122).

Epidermal growth factor receptor (EGFR) belongs to the ErbB receptor family and is involved in cell proliferation and division. Aberrant activation of EGFR is a significant mechanism underlying tumorigenesis and cancer progression (123). Epidermal growth factor receptor pathway substrate 15 (EPS15) is an important substrate of the EGFR pathway. In HCC, EPS15 exhibits high expression levels, which are correlated with tumor cell progression and invasiveness. EPS15-AS1 is an antisense lncRNA of EPS15 that is downregulated in HCC. Increasing the expression of EPS15-AS1 can suppress EPS15 and EGFR, thereby inhibiting the activity of HCC cells (124, 125). In addition, the overexpression of EPS15-AS1 suppresses EGFR and aldo-keto reductase family 1 member B1 (AKR1B1), resulting in elevated levels of free iron and lipid peroxidation in HCC cells. Notably, this inhibitory effect of EPS15-AS1 can be mitigated by the ferroptosis inhibitors ferrostatin-1 and deferasirox (124). AKR1B1 belongs to the aldose reductase superfamily and is involved in the reduction of diverse aldehydes. AKR1B1 is overexpressed in a range of tumors, including HCC (126). Research has demonstrated that EGFR1 inhibition can lead to the upregulation of AKR1B1 and subsequent suppression of the *de novo* GSH synthesis (127). However, the mechanism underlying the upregulation of AKR1B1 remains unclear and requires further investigation.

Zhang et al. identified the role of the lncRNA nuclear paraspeckle assembly transcript 1 (NEAT1) in ferroptosis. NEAT1 is a downstream gene of the tumor suppressor p53. In HCC, erastin treatment promoted the binding of p53 to the NEAT1 promoter and increased the expression of NEAT1 (16). Myoinositol oxygenase (MIOX) is a non-haem iron oxygenase that can catabolize myoinositol to D-glucuronate, and its upregulation can lead to a decrease in the cellular antioxidant capacity and an increase in ROS production (128). NEAT1 competitively binds to miR-362–3p and reduces the interaction between miR-362–3p and MIOX, which promotes MIOX expression and the accumulation of Fe^2+^ and ROS.

**Figure 3:**
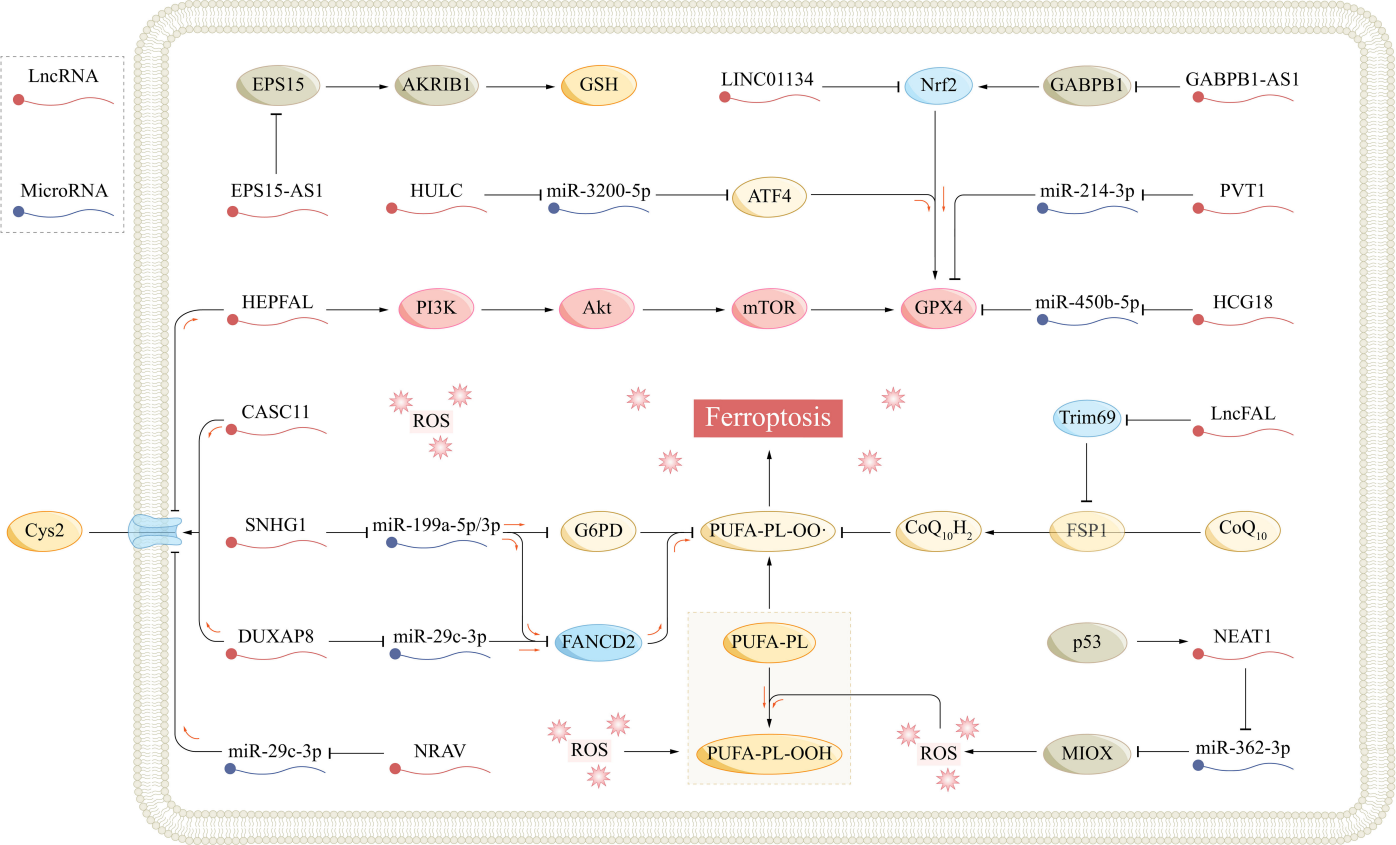
The role of ferroptosis-related lncRNAs in hepatocellular carcinoma. AKR1B1, aldo-keto reductase family 1 member B1; Akt, protein kinase B; AS1, antisense RNA 1; ATF4, activating transcription factor 4; CASC11, cancer susceptibility candidate 11; CoQ10, coenzyme Q10; Cys, cysteine; Cys2, cysteine; DUXAP8, double homeobox A pseudogene 8; EPS15, epidermal growth factor receptor pathway substrate 15; FANCD2, fanconi anemia complementation group D2; Fe^2+^, ferrous; FSP1, ferroptosis suppressor protein 1; FTH1, ferritin heavy chain 1; FTL, ferritin light chain; GABPB1, GA-binding protein B1; G6PD, glucose-6-phosphate dehydrogenase; Glu, glutamate; GPX4, glutathione peroxidase 4; GSH, glutathione; GSSG, glutathione disulfide; HCG18, HLA complex group 18; HULC, highly upregulated in liver cancer; LncFAL, ferroptosis-associated lncRNA; LncRNA, long non-coding RNA; MIOX, myo-inositol oxygenase; mTOR, mammalian target of rapamycin; NCOA4, nuclear receptor coactivator 4; NEAT1, nuclear paraspeckle assembly transcript 1; Nrf2, nuclear factor E2-related factor 2; PI3K, phosphatidylinositol-3 kinase; PL, phospholipid; PUFA, polyunsaturated fatty acid; PVT1, plasmacytoma variant 1; ROS, reactive oxygen species; SLC7A11, solute carrier family 7 member 11; SNHG1, small nucleolar RNA host gene 1.

Activating transcription factor 4 (ATF4) belongs to the cAMP response element-binding protein (CREB) family and is involved in diverse biological processes, such as oxidative stress, endoplasmic reticulum stress, and cell death (129). ATF4 targets numerous ferroptosis-related genes, exerting both promoting and inhibitory effects (129–131). Guan et al. discovered that the lncRNA highly upregulated in liver cancer (HULC) influenced the miR-3200–5p/ATF4 axis via a ceRNA mechanism; the study revealed that the suppression of HULC led to a decrease in ATF4 expression, subsequent inhibition of GPX4 and promotion of ferroptosis in HCC cells (132). In summary, multiple ferroptosis-related lncRNAs are involved in regulating the occurrence, progression, treatment, and drug resistance of HCC, and thus, their study provides a promising potential direction for the treatment of HCC (**Table 1**; **Figure 3**).

**Table 1:** Mechanism of lncRNAs affecting ferroptosis in hepatocellular carcinoma.

LncRNA	Effect	Mechanism	References
PVT1	Inhibit	Inhibiting the miR-214-3p-GPX4 axis and promoting GPX4 expression	(67)
HCG18		Inhibiting the miR-450b-5p-GPX4 axis and promoting GPX4 expression	(106)
CASC11		Directly binding to SLC7A11 mRNA, increasing its stability and half-life	(108)
NRAV		Inhibiting the miR-375-3p-SLC7A11 axis and promotingSLC7A11 expression	(109)
URB-AS1		Promoting punctate distribution and liquid-liquid phase separation of ferritin, suppressing NCOA4-mediated ferritinophagy, and reducing intracellular free iron	(111)
LncFAL		Inhibiting the binding of Trim69 and FSP1 and the ubiquitination degradation of FSP1	(112)
LINC01134		Promoting Nrf2-GPX4 axis and GPX4 expression	(66)
DUXAP8		Suppressing the miR-29c-3p-FANCD2 axis and enhancing FANCD2 expression; promoting the palmitoylation and stability of SLC7A11	(64, 120)
SNHG1		Inhibiting miR-199a-5p/3p and promoting the expression of FANCD2 and G6PD	(122)
HULC		Suppressing the miR-3200-5p/ATF4 axis and enhancing the expression of ATF4 and GPX4	(132)
NEAT1	Promote	Inhibiting the miR-362-3p-MIOX axis and promoting MIOX expression	(16)
HEPFAL		Promoting ubiquitination of SLC7A11, reducing its stability and half-life, and suppressing GPX4 through the PI3K-Akt-mTOR pathway	(63, 110)
GABPB1-AS1		Inhibiting the translation of GABPB1 and the expression of Nrf2	(117)
EPS15-AS1		Inhibiting EPS15 and AKR1B1, suppressing the *de novo* synthesis of GSH.	(124)

AKR1B1, aldo-keto reductase family 1 member B1; Akt, protein kinase B; AS1, antisense RNA 1; ATF4, activating transcription factor 4; CASC11, cancer susceptibility candidate 11; DUXAP8, double homeobox A pseudogene 8; EPS15, epidermal growth factor receptor pathway substrate 15; FANCD2, fanconi anemia complementation group D2; FSP1, ferroptosis suppressor protein 1; GABPB1, GA-binding protein B1; G6PD, glucose-6-phosphate dehydrogenase; GPX4, glutathione peroxidase 4; HCG18, HLA complex group 18; HULC, highly upregulated in liver cancer; LncFAL, ferroptosis-associated lncRNA; LncRNA, long non-coding RNA; MIOX, myo-inositol oxygenase; mTOR, mammalian target of rapamycin; NCOA4, nuclear receptor coactivator 4; NEAT1, nuclear paraspeckle assembly transcript 1; Nrf2, nuclear factor E2-related factor 2; PI3K, phosphatidylinositol-3 kinase; PVT1, plasmacytoma variant 1; SLC7A11, solute carrier family 7 member 11; SNHG1, small nucleolar RNA host gene 1.

## 5 Ferroptosis-related LncRNA and HCC tumor heterogeneity

Tumor heterogeneity is a salient feature of malignancies like HCC. As cancer progress, cancer cells undergo numerous rounds of division and proliferation, triggering molecular biological or genetic alterations that give rise to disparities in growth rate, invasiveness, drug responsiveness, prognosis, and tumor immune heterogeneity (133, 134). Ferroptosis-related lncRNAs play a pivotal role in HCC tumor heterogeneity. For example, NEAT1 has been found to enhance erastin-induced ferroptosis in HCC via the miR-362–3p/MIOX axis (16). In contrast, NEAT1 exhibits high expression levels in non-small cell lung cancer and lung adenocarcinoma, where it acts as an inhibitor of ferroptosis (135, 136). This suggests that ferroptosis-related lncRNAs may contribute to HCC tumor heterogeneity by modulating ferroptosis. However, there are currently limited studies on the differences between ferroptosis-related lncRNAs in HCC and other cancers where ferroptosis is a key mechanism. Future studies will endeavor to unveil the precise mechanisms of these lncRNAs in HCC tumor heterogeneity, thereby offering novel perspectives and approaches for the diagnosis, therapy, and prevention of HCC.

## 6 Conclusion

Ferroptosis, a unique type of PCD, has attracted widespread attention in tumor research in recent years. An imbalance in iron metabolism, lipid metabolism, and the antioxidant defense axis underlies the main process of ferroptosis, which is delicately regulated at the transcriptional, translational, and posttranslational levels. Alterations in iron metabolism are frequently observed in the pathogenesis of tumors. Tumor cells are often characterized by abnormal processes of iron acquisition and utilization, which satisfy their demands for rapid growth and division. This abnormal iron metabolism not only is closely related to the occurrence of ferroptosis but also may influence the tumor growth, invasion, and metastasis. Therefore, targeting ferroptosis in tumor cells appears to potentially be a treatment approach with fewer side effects on healthy cells.

Although lncRNAs do not encode proteins, they play pivotal roles in diverse cellular processes as functional RNAs. In this review, we describe the regulatory roles of lncRNAs in various aspects of ferroptosis and detail the functions of ferroptosis-related lncRNAs in HCC. LncRNAs play crucial regulatory roles in major aspects of ferroptosis, including iron metabolism, lipid metabolism, and antioxidant ferroptosis defense axes. LncRNAs can directly target the expression and stability of crucial ferroptosis-associated factors, such as GPX4 and SLC7A11, or modulate the expression of ferroptosis regulators, such as p53 and Nrf2. More commonly, lncRNAs function as molecular sponges for miRNAs, exerting regulatory functions through the lncRNA−miRNA−mRNA pathways (**Table 1**). Ferroptosis-related lncRNAs play roles in the occurrence, progression, metastasis, and drug resistance of HCC by regulating the levels of free iron, ROS, and MDA in HCC cells. Notably, in related studies, inhibiting ferroptosis in HCC cells consistently accelerated the progression of HCC and led to its resistance to sorafenib, whereas promoting ferroptosis suppressed HCC and increased its sensitivity to sorafenib. Furthermore, several predictive models for clinical prognosis, staging, and immune infiltration in HCC have been centered on ferroptosis-related lncRNAs. According to these models, the lncRNAs in the high-risk group tended to consistently suppress ferroptosis. These results highlight the role of ferroptosis in HCC, demonstrating the potential of targeting ferroptosis in HCC therapy and the feasibility of using ferroptosis-related lncRNA models for predicting HCC prognosis.

Despite the encouraging results achieved, there are still numerous limitations and challenges in the current research. While several models have been constructed through database studies, the lncRNAs utilized in these models are inconsistent, and the predicted patient populations differ, which somewhat restricts their reliability and comparability. More importantly, the experimental validation of these lncRNA markers is still insufficient, making it difficult to precisely determine their functions and impacts in actual patient samples. Further laboratory studies and clinical trials are needed to verify the specific mechanisms of action of these lncRNAs in HCC patients. Additionally, the clinical use guidelines for these clinical prognosis prediction models also require further clarification. We need to understand the applicability, predictive accuracy, and potential limitations of these models in different patient groups. Finally, the roles of serval ferroptosis-related lncRNAs in HCC cell lines have been preliminarily identified, but their accuracy in predicting HCC patient prognosis and immune regulation remains a critical research direction in the future. Currently, while we primarily rely on techniques like gene knockout and overexpression to assess the function of lncRNAs, these approaches may not comprehensively simulate their authentic role in physiological or pathological settings. Furthermore, the intricate nature of lncRNAs poses challenges in thoroughly deciphering their intricate interactions with other biomolecules such as mRNAs and proteins.

Overall, the specific mechanisms of action of ferroptosis-related lncRNAs are crucial for understanding the pathogenesis of HCC. These lncRNAs may have close associations with the expression of ferroptosis-related genes, signaling pathways, or iron metabolism. By studying these mechanisms in depth, we can gain a better understanding of the pathogenesis of HCC, which will provide new insights for the development of treatment strategies. Furthermore, regulating the expression or function of ferroptosis-related lncRNAs offers new potential targets and methods for the treatment of HCC. By modulating these lncRNAs, we can influence biological processes related to HCC, such as proliferation, invasion, PCD, and immunity. In future research, we still need to focus on the following aspects: 1) Develop more precise and accurate methods for studying the functions of lncRNAs, for instance, utilizing single-cell sequencing technology can provide us with a deeper understanding of the expression patterns and functions of lncRNAs in individual cells. Additionally, through high-throughput screening and validation techniques, we can more rapidly identify crucial lncRNAs that are related to HCC. 2) Deeply investigate the specific mechanisms of action of ferroptosis-related lncRNAs in HCC, including their interactions with genes, proteins, or signaling pathways, and how these interactions affect the occurrence and development of HCC. 3) Develop specific regulatory methods targeting these lncRNAs, such as inhibitors or activators, and evaluate their effectiveness and safety in the treatment of HCC. 4) Conduct more preclinical and clinical studies to validate the feasibility and effectiveness of these lncRNAs as therapeutic targets. This includes testing the effectiveness of these treatment methods in animal models and evaluating their safety and efficacy in human clinical trials. By deeply studying the mechanisms of action of ferroptosis-related lncRNAs and developing specific therapeutic methods targeting them, we have the potential to provide new insights and strategies for the treatment of HCC.

## Author contributions

HC: Writing – original draft. ZH: Writing – original draft. JS: Investigation, Writing – review & editing. XS: Investigation, Writing – review & editing. QM: Investigation, Writing – review & editing. YL: Investigation, Writing – review & editing. ZR: Investigation, Writing – review & editing. XL: Investigation, Writing – review & editing. RM: Writing – review & editing. YW: Writing – review & editing. DL: Writing – review & editing.
